# Comparative Diagnosis of the Urban Noise Problem from Infrastructural and Social Sensing Approaches: A Case Study in Ningbo, China

**DOI:** 10.3390/ijerph19052809

**Published:** 2022-02-28

**Authors:** Yutian Si, Liyan Xu, Xiao Peng, Aihan Liu

**Affiliations:** 1 College of Architecture and Landscape Architecture, Peking University, Haidian, Beijing 100871, China; 1701214565@pku.edu.cn (Y.S.); pkupengxiao@pku.edu.cn (X.P.); aliu685@gwu.edu (A.L.); 2 Sichuan Territorial Planning Institute, Chengdu 610081, China; 3 Department of Data Science, The George Washington University, Washington, DC 20052, USA

**Keywords:** urban noise, infrastructure sensing, social sensing, acoustic environmental quality standard, “high-frequency” city, Ningbo

## Abstract

Urban noise causes a variety of health problems, and its prevention and control have thus become an important research topic in urban governance. Although existing literature is fairly comprehensive in revealing the physical noise patterns, it lacks the concern of people’s perceived seriousness, especially at the macroscopic, i.e., citywide scale. In this paper, we borrow from the “exposure-perception-behavior” theory in environmental psychology, and propose an analytical framework for diagnosing the urban noise problem that integrates the Infrastructural and Social Sensing perspectives. Utilizing noise monitoring data that fills the spatiotemporal granularity gaps of official noise monitoring, as well as the “12345” urban complaint hotline records which serve as a proxy for residents’ perceived noise levels, we empirically examine the mechanisms for physical magnitude and perceived seriousness of urban noise, respectively, by taking the Jiangbei District of Ningbo City, China as an example. Results show that the existence of perceptual bias and behavioral preference effects did shape people’s *perceived* noise problem map that is vastly different from that of the *physical* noise magnitude, in which the semantics of urban places, temporal rhythms of life, and population demographics significantly influenced people’s tolerance of noise. We conclude the paper with suggestions on updating the existing National Standard for urban noise regulation to reflect the perceptual aspect, and also methodological discussions on possible ways to recognize and utilize the perceptual bias in social-sensing big-data to better accommodate urban governance.

## 1. Introduction

Noise poses numerous threats to people’s physical and psychological health and is an intrinsic part of environmental pollution [1]. The problem of noise pollution is particularly acute in cities of developing countries due to their limited urban governance capacity [2,3,4,5]. In China, for example, the *2013 China Ecological Environment Survey* reported that noise is one of the four major pollutants in China [6]. The compliance rate to the National Acoustic Environmental Quality Standard (GB 3096-2008) [7] (*National Standard* henceforth) of urban noise has been low. In 2020, noise complaints accounted for 41.2% of all environmental-problem-related public complaints, assuming a second place among all pollutant sources [8]. The various negative effects of urban noise have prompted extensive research in the field of built environment. Currently, studies on the noise patterns of the built environment have become fairly established at the mesoscopic (blocks, plazas, and parks) and microscopic (buildings, indoor environments) scales [9,10]. In terms of noise governance, there is also an abundance of studies on noise reduction technologies, management strategies, and noise source control at small and medium scales [11,12,13,14]. Based on these academic progresses, the International Organization for Standardization (ISO) has formally defined the concept of the soundscape [15], and some cities in advanced economies have even established institutionalized environmental noise control programs through soundscape-mapping tools such as the acoustic map [16,17]. However, at the macroscopic, i.e., citywide scale, there is still a lack of research on fine-granularity spatiotemporal patterns, occurrence mechanisms, and governance strategies of the noise problem [13,18,19,20], and it is again especially so in developing countries [3].

This situation is largely caused by the lack of empirical observation data on urban noise at the appropriate spatiotemporal granularity. Traditional urban noise studies tend to rely on direct monitoring of noise as the data source. In other words, they follow an infrastructure-sensing approach. Admittedly, direct monitoring instruments can objectively reflect the magnitude of noise. China’s *National Standard* on urban acoustic environmental quality (Table A1 in Appendix A), like those of other countries and international organizations [15], is based on the physical magnitude of noise. However, one prominent difficulty in the implementation of this standard lies in the noise monitoring itself. Noise monitoring data are incomplete in most Chinese cities in spatiotemporal granularity terms, which means (1) the small number of sampling points, which causes the severe lack of coverage of urban space, and (2) the fixed sampling time, which lacks reflection of the temporal variation of noise magnitude. Taken together, existing noise monitoring data are sparse in space and time, thus reflecting a rather coarse-grained and static urban noise landscape. However, modern cities bear a fast life rhythm, which requires the “high-frequency”, or spatiotemporally fine-grained observation of the city for researchers [21], as in the case of noise research. As a result, the coarse-grained, static noise landscape described above is not sufficient to reflect the full picture of urban noise, and is therefore inadequate to support the governance of urban noise in a precise and responsive manner. Although scholars have developed methods to infer overall, dynamic noise levels from local, static data [22], such inference is not a substitute for observation, and is often difficult to validate. Overall, considering its extensive requirements in terms of equipment, manpower, and other inputs, empirical observation of urban noise at high spatiotemporal granularities is still a challenging task.

**Figure 1:**
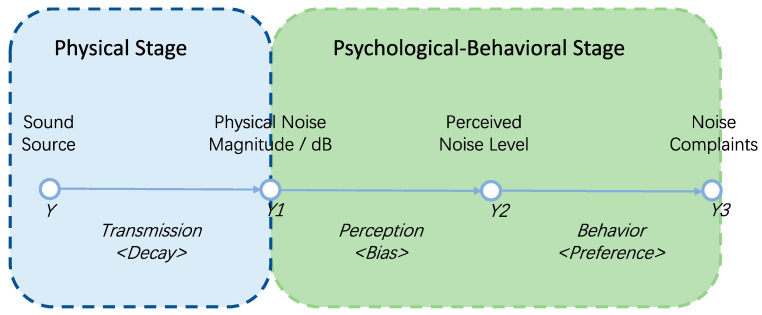
A conceptual framework for understanding the urban noise problem.

Notwithstanding the difficulties in dynamically monitoring urban noise, noise control under the urban governance perspective does not necessarily require the extreme ideal of real-time noise monitoring. This is because the objective of urban governance is people’s subjective satisfaction of the acoustic environment, which is not the same thing as the objective, physical magnitude of noise. Indeed, stemming from the “exposure-perception-behavior” framework in environmental psychology [23], we can establish a framework for conceptualizing the urban noise problem from the governance perspective (Figure 1), which includes two stages. The first is a physical stage, where the noise generated by certain sound sources is transmitted to various places through certain pathways and a distance-decay process. After entering the human ear, there comes the second stage, a psychological-behavioral one, where people sense to form their perception of the noise, and some of them may decide to complain about it. For the second stage, studies have shown that different people have different “thresholds” for the noise to trigger negative feelings, and that people’s tolerance of noise (also referred to as “annoyance”) is not simply linearly related to physical magnitude of noise [24,25,26,27]. Rather, people’s perception of noise are influenced by the environment [28,29], place semantics [14,30,31], and their own demographic characteristics [32,33] in complex ways. Apparently, only when people find it intolerable to abide the noise will they choose to take actions, or putting it another way, to complain. 

Technically, in the framework of Figure 1, direct observation of fine-granularity noise magnitude when reaching the human ear (Y1) is difficult to obtain; direct observation of people’s perception of noise (Y2), however, is even more so as it is a state in the human mind and is impossible to “read”. These challenges make it difficult for researchers to infer people’s preference or tolerance for noise (Y3) from the chain of analysis in Figure 1. Nevertheless, the advance of Information and Communication Technology (ICT) and other technologies in the field of urban governance in recent years has opened up new opportunities to study the urban noise problem. Supported by new data sources such as Internet User-generated Contents (UGC), or records in e-government platforms, researchers are now able to utilize proxy data on people’s stated preferences on urban events. In the case of urban noise, the “12345” urban complaint hotline, which is commonly established in Chinese cities, is an excellent proxy to observe people’s noise preferences. The “12345” hotline is roughly equivalent to the “311” hotline in United States cities, through which citizens can complain about various problems in everyday urban lives, and noise is one of the most complained issues on the hotline. Therefore, through the noise complaint records on the “12345” hotline, we can observe people’s expression of noise preference. Methodologically, such observation is a reflection of the emerging “Social Sensing” approach, where, with the aid of the ICT, each person can act as a sensor of social events, generating data with individual markers and spatiotemporal semantic information anytime and anywhere [34,35]. At the collective level, we can use Social Sensing data to reveal the spatiotemporal patterns of the observed objects for this paper, noise, and investigate the mechanisms by which they occur [36]. Such analysis helps us to understand the rationale of urban residents complaining about noise. And further, by comparing this rationale with the physical occurrence mechanisms of noise generation and transmission, we can better understand the relationship between physical and psychological processes of the noise problem, and develop effective urban noise control policies, which would be of great importance in urban environmental governance.

In summary, we conduct noise monitoring with improved spatiotemporal granularity in this study, and compare it with noise complaint records from the “12345” hotline data, which allows us to investigate the following two research questions:

(1) What are the fine-grained spatiotemporal patterns of objective, physical magnitude of noise, as well as the residents’ subjectively perceived seriousness of noise in typical urban settings? And what similarities and differences the two pictures show?
(2) What are the mechanisms that gives rise to the physical and perceptual noise landscape, respectively? And what similarities and differences the two mechanisms have?

In this paper, we take the Jiangbei district of Ningbo City as an example, and examine the above questions. The rest of the paper is organized as follows. Section 2 describes the methods and data. Section 3 presents the empirical findings on the spatiotemporal patterns and mechanisms of the monitored physical magnitude of noise and the perceived seriousness of noise. We conclude the paper in Section 4 with a summarizing conclusion, and discussions on the methodological and practical implications of the research.

## 2. Materials and Methods

### 2.1. Study Area and Data Sources

#### 2.1.1. Study Area

**Figure 2:**
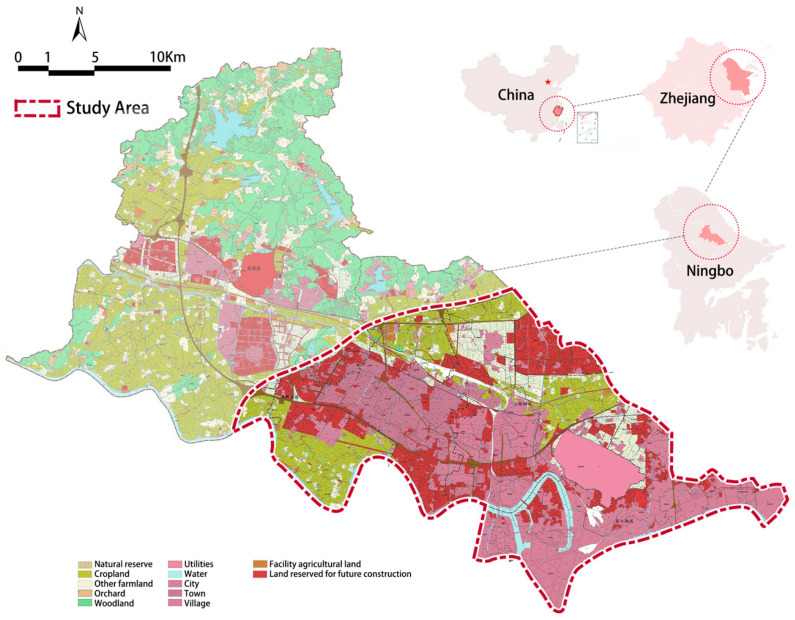
Study area.

Jiangbei District, Ningbo City, China, was selected as the case city for this study (Figure 2). Specifically, we chose the urban and peri-urban areas with a high concentration of population in the district, i.e., the area east of the bypass highway as the boundary of the study area. The area is 102 km^2^ in size and with a population of about 400,000 by the end of 2019. Ningbo is an important port city in East China, and the study area covers the central business district, the old town area, and an entire urban-rural gradient, where major types of urban noise environments are included, making the area a representative case for studying the urban noise problem.

#### 2.1.2. The “12345” Data

The “12345” urban problem complaint hotline is an important part of China’s digital urban governance system, providing a bottom-up feedback channel for addressing urban problems of all sorts. The complaint records in the study area were provided by the Ningbo Bureau of Urban Management for the period from 1 January 2017 to 31 July 2019, and the sample size is 1116. The record data include the time, location, content, handling status, and handling government department of the complaints, and were anonymized as necessary before being provided to the researcher. A sample of typical noise complaint records is shown in Table A2 in Appendix B. According to the noise types involved in the complaints, the complaint records can be divided into two categories: everyday life-related noise (such as dog barking, noise from streetside restaurants, loud advertisement broadcasting, traffic noise, etc.), and construction-related noise.

#### 2.1.3. Noise Magnitude Data Acquisition and Processing

We obtained the noise monitoring data from government information disclosure by the Ningbo Ecological Environment Bureau. The data include two types of records: (1) noise monitoring data for the acoustic environment functional areas in the study area. This record has only one sample point in the whole area, and is continuously monitored with the fixed-point monitoring method as stipulated by the National Acoustic Environmental Quality Standard, and the data obtained are the average day and night equivalent sound level values for the whole year of 2019; (2) the regional acoustic environment monitoring data in the study area, which contains 23 monitoring points. For each monitoring point, one day (non-holiday) which meets the general outdoor noise monitoring conditions is chosen to conduct a 10 min noise level monitoring, and the monitoring is conducted once a year.

**Figure 3:**
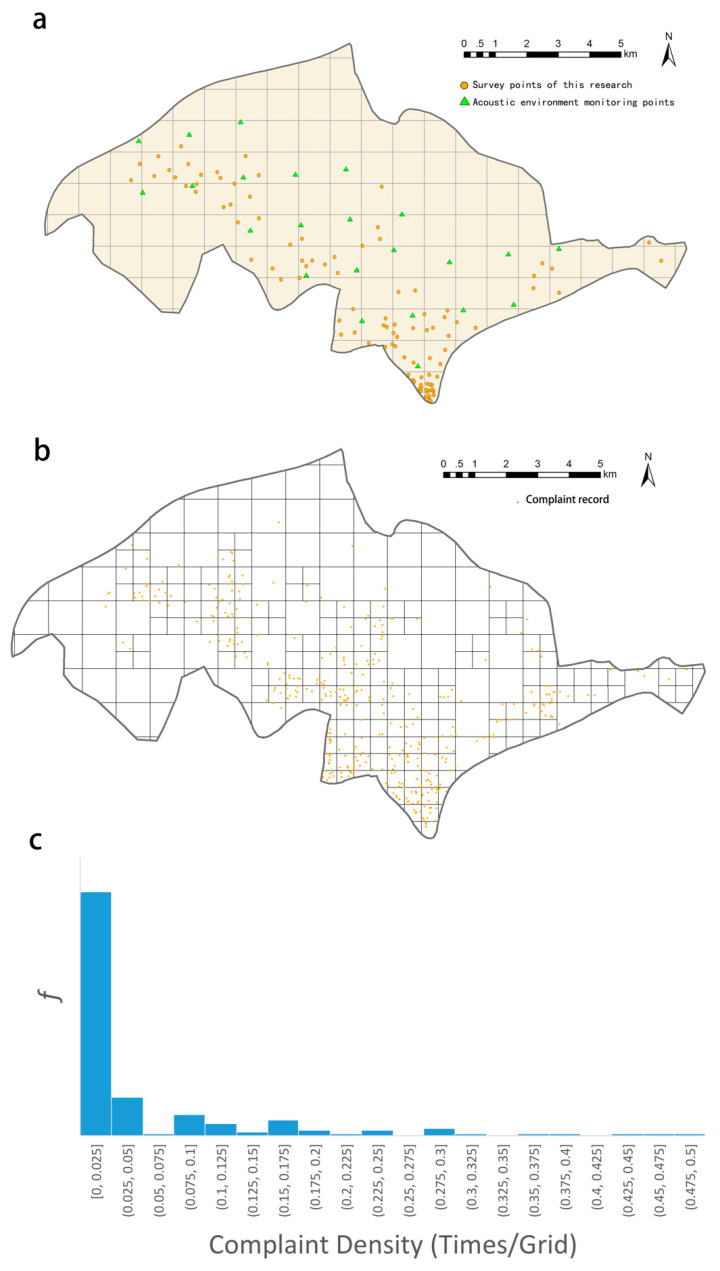
Analysis grids and noise monitoring and complaints. (**a**) noise monitoring grids and sampling locations; (**b**) analysis grids and noise complaint locations; (**c**) histogram of the everyday life-related noise complaints in the analysis grids.

Since the above officially provided noise monitoring sampling is very sparse in both spatial and temporal terms, in this study we conducted field noise monitoring under standard measurement conditions to obtain noise magnitude data at higher spatiotemporal granularity. For the entire study area, we first designated a regular grid system of 1 × 1 km (see below for details), and then determined the number of monitoring points needed within each grid through stratified sampling based on the number and distribution of the “12345” noise complaint records in each grid, such that the ratio of number of complaint records to the number of noise monitoring points to be 10:1. Given the number of sampling points (107 in total), random points were generated as locations for noise monitoring (Figure 3a). We used the AWA6228+ sound level meter for sound monitoring, which complies the National Standards of GB/T3785-2010 (IEC61672) Level 1 and GB/T3241-2010 (IEC61260) Level 1; and used the AWA6201 sound calibrator, which complies the National Standard GB/T 15173-2010 Level 1 [37]. All instruments, as well as the monitoring methods used were in accordance with the specifications of the *National Standard*, and the latitude and longitude of each sampling point were recorded at the same time with a portable Global Navigation Satellite System (GNSS) device. The noise monitoring records at each sampling point included the sound pressure LA, which were recorded once per second, and were converted to equivalent continuous A-weighted sound pressure level ($L_{aeq}$) according to the method in the *National Standard* [7]. The measurement reflects the energy average of A sound level (in dB) during the specified measurement time *T*. Its calculation formula is:

$$L_{aeq}=10\mathrm{lg}\left(\frac{1}{T}*\int_{0}^{T}10^{0.1LA}dt\right)$$

The noise measurements obtained from the above process were imported into ESRI ArcGIS (ESRI, Redlands, CA, USA) together with the official data of 23 effective regional acoustic environment monitoring points, and the distribution map of noise magnitude were obtained by inverse distance weight interpolation. The principle of the inverse distance weight interpolation method is most consistent with the attenuation characteristics of noise transmission, i.e., when noise propagates in the atmosphere, the magnitude decays inversely proportional to the distance squared as the propagation distance increases, and therefore most noise studies have adopted this interpolation method [2,38].

#### 2.1.4. Other Data Sources and Calculation of Indicators

(1) Land use data

The land use data (for the year of 2018) of the study area used in this study were obtained from the Open Data of Tsinghua University (EULUC-China). This data has an average precision of 65.7% and a maximum precision of 82.9% on the primary classification and an average precision of 60.2% and a maximum precision of 80.0% on the secondary classification, and thus has a high degree of confidence for research purposes [39]. We manually corrected this data with the status quo satellite maps, and finally obtained a land use map including 6 primary land use categories and 13 secondary categories (Table A4 in Appendix D).

(2) POI data, and the city functional mixture index calculation

The POI (Point of Interest) data used in this study were obtained from the online map service Gaode Maps (Available online: www.amap.com; accessed on 7 September 2019), which classifies the POIs into eight functional categories, namely, food and beverage services, leisure and entertainment services, financial services, government and administration agencies, office buildings, shopping services, tourist attractions, and automobile services. Besides, in order to examine the relationship between street vending activities which might inflict everyday life-related noise complaints, we also extracted the complaint records from the “12345” hotline about street vending as a supplement to the POI data.

Further, to reflect the semantic differences of urban places, this study introduces an urban functional mixture index based on land use or POI composition of a place. The index is calculated based on the principle of entropy in information theory, and its value is related to the total number of land use or POI types in a grid and the percentage of each type. The calculation formula is as follows.

$$S=-\overset{n}{\underset{i=1}{\sum }}P_{i}\log_{10}P_{i}$$

where *S* is the information entropy, which represents the degree of functional mixture of an urban place. The higher the entropy value, the higher the degree of mixing of urban functions [40]. Taking POI as an example, the higher the entropy value of POI, the more diverse and complete the types of services that this grid can provide, and the smaller the possibility that the grid is dominated by a certain POI type; *n* denotes the number of land use or POI types in a certain grid; *P_i_* denotes the proportion of a certain type among all types. Based on spatial statistics of each grid with GIS tools, *n* and *P_i_* can be obtained to calculate *S*.

(3) Mobile phone signaling data

The mobile phone signaling data used in this study were provided by the telecom operator China Unicom. Mobile phone signaling data record cell phone users’ power on, power off, calling, being called, sending and receiving SMS, switching base stations, and periodic location update events. The data provided to us contain anonymized user IDs, the time of the above events, the base station number where they were located at that time, and the geographic coordinates of the events, which were recorded according to the base station triangulation method with an accuracy of about 200 m. China Unicom is one of the four major operators in China. With a market share of 21%, it constitutes a large sample of the entire population; at the same time, the data provider has already performed resampling of the data for correction of potential bias caused by people without cell phones, such as children, so its mobile phone signaling data can be considered as an unbiased sampling of the population and can be used to describe the spatiotemporal characteristics of residents’ activities such as living, working, and recreation [41]. In this study, the basic demographic and behavioral characteristics of local residents in the study area were profiled by three types of stay and five age groups as provided by the data. The three stay types include residence, work, and visiting, and their identification is based on the logic of the users’ common life rhythms (Table A3 in Appendix C). Among them, the residence and work tags are determined based on the nighttime and daytime stay patterns of cell phone users over a long period of time, with each user having at most one residence tag and one work tag. The visiting tag, on the contrary, is recorded as a visit as long as the user has a stay of more than 30 min at a location, with no upper limit on the number of visits. To reflect the necessary stable spatiotemporal patterns, one month of user data was used for the above stay type tagging in this study.

#### 2.1.5. Analysis Grids

This spatiotemporal analysis in this study is based on a regular grid. First, a preliminary grid division of 1 × 1 km was carried out for the study area. However, comparing this grid with the “12345” noise complaint records, it can be found that its spatial granularity is too coarse to reflect the spatial heterogeneity at a smaller scale in some areas with intensive complaints. Therefore, a finer granularity of the grid is necessary. However, there are areas with rather low density of human activities such as large agricultural fields at some marginal locations in the study area, and such areas are not suitable for further spatial division because it would unnecessarily obscure the focus of the analysis and would also increase the zero values in the final analysis data, which would have side impacts on the accuracy of the parameter estimation of the statistical model. We believe that since people are the main subject of urban noise problems, the appropriate overall study area is the area of the city with human activities, and therefore samples with a significant lack of human activities should be avoided as much as possible to avoid sampling bias. Based on the above considerations, we subdivided the grid with ≥4 complaint points on the 1 × 1 km grid into a 500 × 500 m grid. The obtained analysis grids are shown in Figure 3b, which yield a total of 278 grids as the basic spatial analysis units of this study. It should be noted that in the statistical modeling later on, we excluded the grids with zero POI and complaint records, so a total of 210 analysis grids were finally involved in the statistical modeling.

### 2.2. Analysis of Spatiotemporal Patterns of Noise Magnitude and Complaints

According to the time tag of the “12345” complaint records, we can statistically get the changes of the volume of the “12345” complaints in different time periods. The annual day and night equivalent sound level averages provided by the Ningbo municipal government present the continuously monitored noise magnitudes throughout the year. The records are compared with the number of complaints, which reflect people’s noise preferences at respective time periods. Similarly, comparing the interpolated noise magnitude map and the noise complaint kernel density map, we can examine people’s noise complaint preferences in different locations. In general, four scenarios exist in terms of the relationship between noise magnitude and noise complaints in both spatial and temporal senses: (1) high noise magnitude, high complaint volume; (2) high noise magnitude, low complaint volume; (3) low noise magnitude, high complaint volume; and (4) low noise magnitude, low complaint volume. Among them, of particular importance are scenarios (2) and (3), as both appear counter-intuitive.

### 2.3. Explanatory Model for Physical Noise Levels

We then constructed the model for explaining the physical noise magnitudes with built and social environment factors. The dependent variable in the model is the average of all monitored noise magnitudes during the study period, where each 10 min sound monitoring result at a sample site was converted to an $L_{aeq}$ value following the method in the *National Standard* [7]. The rationale for selecting independent variables is based on the basic principles of acoustics, i.e., considering both noise source and transmission path factors [29]. Nevertheless, in the absence of direct noise source observation, we opt to use proxies for both noise source and transmission path feature as the independent variables, including population density, built-environment features, and urban place semantics (Table 1). As the dependent variable and all independent variables are continuous, we fit the model using multiple linear regression, a common practice in most literature on the acoustic environment [42,43]. The model takes the form of:

$$y=\beta_{0}+\beta_{1}x_{1}+\ldots +\beta_{k}x_{k}+\varepsilon$$

where $x_{1}$, …, $x_{k}$ are the independent variables; *y* is the dependent variable; $\beta_{0}$, …, $\beta_{k}$ are the regression coefficients; and ε is the error term.

### 2.4. Explanatory Model for Perceived Noise Levels (Complaints)

For the explanatory model for people’s perceived noise levels as proxied by complaint records, we first need to limit the scope of the model. On the one hand, compared to the irregularly generated construction noise, the everyday life-related type of noise is more structural in nature for cities. On the other hand, due to limitations in research conditions, we only monitored the noise magnitudes during the daytime, and thus a meaningful comparison can only be conducted within the time period. On balance, we only chose the everyday life-related type of noise complaints as the dependent variable, and constructed an explanatory model for the daytime, i.e., the period from 6:00 to 19:00. In general, this time period includes a variety of everyday life states such as people’s commuting, social out, and home stay, which cover typical occasions when the everyday life-related type noise complaints may occur, and thus can generally reflect the long-term, structural characteristics of people’s perception and preference statement (complaint-making) of urban noise.

The independent variables in the model include the physical noise magnitude, as well as various demographic and urban place semantic factors that may affect the two phases of noise perception and behavior in Figure 1. They include land use, POI, functional mixture indices of urban places, and demographics of the working, living, and visiting populations as recorded in the mobile phone signaling data (Table 2). Among them, the land use and POI factors reflect the semantics of urban places at different spatial granularities, and we also added the functional mixture indices to reflect the diversity of place semantics. For similar considerations, the various types of people under the three activity labels are also included to reflect ways people using urban places.

We used the number of noise complaints within each analysis grid as the dependent variable. The histogram of the variable for the 210 samples (Figure 3c) demonstrated that a large percentage of these observation values are 0, where no noise complaints occur in the corresponding grids. The many 0′s in the dependent variables means that the variable is truncated at 0, which requires the use of the Tobit model to address the truncated data. When the left-hand side constrained point is 0 and there is no right-hand side constrained point, the Tobit model introduces the latent variable $y^{*}$, which is linearly related to the independent variable. Since we can only observe in the sample $y=\max\left(0,\;y^{*}\right)$, the Tobit model is set as follows:

$$y_{i}^{*}=X_{i}^{\prime }\beta +u_{i}$$

$$u_{i}\sim N\left(0,\sigma^{2}\right)$$

$$y_{i}=\{\begin{array}{c} \;y_{i}^{*},|y_{i}^{*}>0\\ 0,|y_{i}^{*}\leq 0 \end{array}$$

When the latent variable $y^{*}$ is less than or equal to 0, the dependent variable y is equal to 0; when $y^{*}$ is greater than 0, y is equal to $y^{*}$. Meanwhile, we assume that the perturbation term $u_{i}$ follows normal distribution with a mean of 0 and variance of $\sigma^{2}$. For the case in this study, this model can be interpreted as the non-zero observations in the sample are the grids where complaints have already occurred, i.e., $y_{i}=y_{i}^{*}>0$. However, this does not mean that noise has no effect on the grids where complaints have not yet occurred, i.e., $y_{i}=0,\;\left(y_{i}^{*}\leq 0\right)$. Lastly, for parameter estimation, since the commonly used OLS estimation method is a linear regression of the whole sample, where the nonlinear perturbation term will be included, and will lead to inaccurate parameter estimation, the Maximum Likelihood Estimation (MLE) method is used to estimate the model parameters. All models are fit with the Stata statistical package.

## 3. Results

### 3.1. Spatiotemporal Patterns of Physical Noise Magnitude and Noise Complaints

The year-round mean diurnal equivalent sound levels in the study area were 55.9 dB during the day (07–21 h) and 51.3 dB at night (22–06 h the next day). However, the number of the “12345” noise complaints was much higher at night than during the day, 310 and 768, respectively, showing an opposite correlation with the physical noise magnitudes.

**Figure 4:**
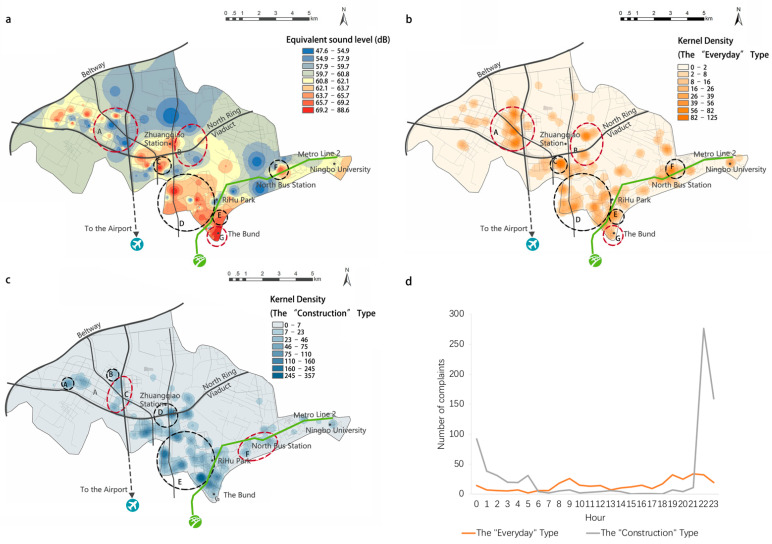
Spatiotemporal distribution of monitored and complaint noise records. (**a**) Kernel density of monitored noise levels; (**b**) kernel density of noise complaints (the “everyday” type); (**c**) kernel density of noise complaints (the “construction” type); (**d**) time series of noise complaints within a day.

An examination of the number of different types of noise complaints during the day shows more details of its temporal patterns (Figure 4d). On the one hand, there are more everyday life-related noise complaints than construction-related ones during the daytime. On the other hand, although there is a small increase in the everyday life-related noise complaints at night in absolute terms, there are far more construction-related noise complaints than everyday life-related ones at night. In particular, the daily peak of complaints after 22:00 is overwhelmingly contributed by construction-related noise complaints.

We then consider the spatial patterns of physical noise magnitudes and noise complaints. Figure 4a,b compares the physical noise environment map (a) and the noise complaint map of the everyday life-related type of noise (b). It is obvious that the hotspot areas of the two have significantly different distribution patterns. In some areas such as C, D, E, and F which are marked with black circles, noise magnitudes are high, and noise complaints are also frequent, such that the two show some form of positive correlation. However, in other places, such as areas A, B, and G which are marked with red circles, it can be seen that their noise magnitudes and complaint numbers do not seem to show positive correlations. Particularly, both areas A and B have very low noise levels, yet they have intensive complaints. On the contrary, the number of complaints in area G, which has the highest physical level of noise in the whole study area, does not seem to be high. 

Similarly, Figure 4a,c compares the physical noise environment map (a) and the noise complaint map of the construction-related type of noise (c). The number of complaints in the areas within the black circles, such as area E, is roughly positively correlated with physical noise levels. This is intuitively understandable as this old town area has been undergoing urban revitalization, and the noise from the large building and metro construction sites may have annoyed people nearby. In contrast, areas A, B and D are distributed with industrial parks, small factories, etc., which tend to produce long-time noise, and have indeed generated a high volume of complaints. However, areas C and F, marked with red circles, have lower noise levels, yet there are also a large number of complaints for construction noise. Among them, area C is largely a residential area, while area F is a university town. 

### 3.2. Mechanisms for the Forming of the Physical Noise Environment

The results of the model for the physical noise environment are shown in Table 3. The model is statistically valid overall (F = 13.051, *p* = 0.000). The VIFs of all variables in the model are less than 10, indicating that there is no covariance between the independent variables, and the model fit is reliable.

Results show that about 41% of the noise magnitude (equivalent noise levels) can be explained by the population and POI factors (Adjust-R^2^ = 0.409). Population density contributed the most to noise magnitude. The higher the population density within a grid, the higher the equivalent sound level, which is in line with the common sense that crowded places are noisier. Regarding the POI, or urban place semantic factors, shopping services, office buildings, leisure and entertainment services, government and administration agencies, financial services, and tourist attractions all have different degrees of influence on the equivalent sound level. Specifically, the more office buildings, leisure and entertainment services, government and administration agencies, and tourist attractions, the stronger noise; the more shopping services and financial services, the less noise. For the latter, we speculate that this is because of the fact that the vast majority of shopping service and financial service POIs in the study area are actually community-level outlets, and thus they actually become effective proxies for residential communities, which generally do indicate lower noise levels. It is noteworthy that the POI functional mixture index is not significant, indicating that the richness of the types of services in the study area has no direct effect on the equivalent sound level.

### 3.3. Mechanisms for the Occurrence of Noise Complaints

The results of the model for the occurrence mechanism of noise complaints are shown in Table 4. The model is statistically valid with a *p*-value of 0.000. 61.5% of the complaints can be explained by the independent variables (Pseudo R-squared = 0.615), and the partial marginal effects of the variables are given in Table 5.

Based on Table 4 and Table 5, in the case of *y*|*y* > 0, i.e., in the sample of grids with the number of complaints greater than 0, the average marginal contribution of the physical noise magnitude (equivalent sound level) is 0.005. This is to say, each 1 dB increase in noise magnitude increases 0.005 complaints per unit area (grid). In comparison, each 1-fold increase in the proportion of residential land use increases 0.107 complaints per unit area, and each 0.1 increase in land use mixture index increases 0.006 complaints per unit area. For the demographic variables, the marginal effect on noise complaints is negative for all residential groups from 19 to 64 years old, while a positive marginal effect of 0.262 is generated for the working group from 35 to 49 years old. Apparently, the working population in specific age groups is likely to be an important component of the complainants for daytime, everyday life-related noise. Overall, the mechanistic model of noise complaints suggests the following picture: while increased ambient noise magnitude does push up the number of complaints, the place semantics and demographic characteristics of the city significantly distort the simple positive correlation between physical noise levels and volume of complaints, thus validating our hypothesis that people’s subjective perception, as well as their individual preference on noise would give rise to a different picture of the noise problem with what a physical noise map would indicate.

## 4. Discussion

The findings reveal the spatiotemporal patterns of the physical and perceived noise landscapes, most of which are subject to intuitive interpretations. For example, regarding the spatiotemporal heterogeneity of the physical and perceived noise as found in Section 3.1, the dramatic increase in noise complaints at nighttime might be explained by the probable stronger negative emotions caused by noise compared to those in daytime, considering the negative effects of noise on sleep such as increased wakefulness, reduced sleep length, fragmented sleep cycles, and reduced deep sleep [44,45,46]; also, an intuitive explanation of the spatial inconsistency between the noise magnitude and complaints as shown in Figure 4a,b is that areas A and B are largely residential areas, while area G is a bar district. The difference in urban function between the two may have led to people’s different expectations of the quietness of the acoustic environment, and thus they show different levels of tolerances to noise. The same rationale may also apply in interpreting the pattern differences as shown in Figure 4a,c.

Further, through a comparative analysis of the occurrence mechanisms of the physical noise magnitude and the perceived noise seriousness as proxied by people’s noise complaint records, this research relates physical noise to people’s perception and preferences regarding noise, thus providing an alternative, psychological angle to understand the urban noise problem that complements existing practices, which characterize urban noise environments purely in physical terms. Our findings uncover the structural difference between the physical and perceived noise landscapes in both spatial and temporal terms. Temporally, the acoustic environment in the study area is quieter at night than during the day overall, but there are far more noise complaints at night than during the day. Spatially, noise complaints occur not only in areas which are physically noisier, but also in areas where people’s subjective factors including demographic attributes and place semantics may induce complaints about noise despite a quieter environment in physical terms. Particularly, we find specific factors that might contribute to the soar of noise complaints, such as people of certain age groups (over 50) or urban places of certain land-use types (residential), which are in accordance with the “common sense”.

### 4.1. Necessity for Inclusion of the Social Sensing Perspective in Urban Studies to Fill the Grunalarity Gap

These findings confirm that traditional sensors, such as noise monitors, can only obtain objective decibel values of noise and yet lack a human-centered value concern. This, coupled with their generally high cost, makes traditional sensing approaches significantly limited in their ability to directly address people-related urban problems. The Social Sensing approach, on the contrary, by using the people as “sensors”, is less costly in terms of data collection, and are advantageous in observing the city states which are more human-focused. Therefore, the Social Sensing approach can serve as an “information bridge” for urban science and planning disciplines, and it is especially so under the “high frequency” city perspective. In the past, when data were scarce, people would focus more on large-scale changes in cities over long periods of time, with little attention paid to high-granularity dynamics [21,47]. The introduction of the socially-sensed data can fill this gap, as they provide urban planners and managers with perspectives that might otherwise be hidden or too obscure to detect. One support of this argument is that our noise perception model, which used one Social Sensing data source (complaints) as the proxy for people’s noise perception, actually yields better explanation power than much previous research which utilized direct survey data on noise perception in that ours has a considerably higher R^2^ [9,48,49]. Thus, the introduction of the Social Sensing data may help bridge the gap between macro-scale knowledge and specific phenomena, and have great potential for application in urban studies, as well as planning and governance practices [35].

### 4.2. Turning the Self-Sorting Bias in Social Sensing Data into Useful Governance Tools through the “Nudging” Strategy

One methodological issue that is worthy of discussion, though, is the potential bias inflicted by the nature of the Social Sensing approach. Researchers have found that Social Sensing data are often biased in terms of demographics and spatiotemporal distribution, and thus do not reflect the full picture of the observed objects [50,51,52]. However, this paper proposes an alternative way of interpreting, and even exploiting, such biases enabled by the decomposition of the sources of biases in Social Sensing data. Based on the framework in Figure 1, the generation of Social Sensing data actually consists of two stages, namely, the stage of perceiving objective reality, and the stage of acting based on the perceived outcome. The first of these stages generates perceptual bias, which may be due to differences in the perceptual abilities of the perceiver, or to sampling bias of the perceiver relative to the overall population. Fortunately, by statistical principles and techniques, both biases can be controlled in applied research. For the former, as the distribution of basic sensory perceptual abilities is approximately normal in the population [53], the bias can therefore be controlled for by large sample sampling, which is usually not an obstacle given the big-data nature of Social Sensing data. For the latter, it can be corrected by resampling the sample [54], or by synthetic population construction [55] techniques, when the joint distribution of the control variables is known for the overall population. 

As for the second stage, since the reactions based on perceived outcomes (in this paper, it refers to people’s behavior of making complaints) are made by the perceivers based on their behavioral preferences, the bias here undoubtedly arises from the self-sorting effect in such decisions. Admittedly, this bias cannot be simply eliminated with statistical techniques. However, in certain circumstances where the patterns and mechanisms of such self-sorting effects *per se* are the central concern, the bias is not a nuisance but rather an advantage that can be exploited as it presents exactly what is needed: the characteristics of people’s behavioral preference. For example, from the perspective of urban governance, it can be argued in a sense that those noise problems that are complained about have a high priority for resolution. In other words, for the governance of the urban noise problem, people’s subjective perception of noise and their consequent behavior (complaints) may be as important as, if not more important than, the objective, physical magnitude of noise. Hence, we are brought to the context of “nudge” theory [56], which states that people’s “predictable irrationality” can be utilized to serve certain policy purposes. In the context of this research, people’s “predictable irrationality” is reflected in the fact that they appear to care more about the *perceived* rather than the *physical* noise level, and the former is precisely what the Social Sensing data, such as complaints, can record. Thus, through proper understandings of the former, one can accordingly design policies with typical “nudge” techniques, such as information exposure and feedback provision to encourage people’s complaint activities, such that the hot spot of noise problems can be located and measures be taken subsequently. In a broader sense, in any research with Social Sensing data, as long as the cognitive-behavioral process involved conforms to the framework in Figure 1, the data can be decomposed with the above-mentioned methods for bias control and self-sorting effects observation. Thus, the research framework of this paper has implications for a wide range of research topics, as well as urban governance practices in general.

### 4.3. Policy Implications: Update of the National Acoustic Environmental Quality Standard, and Beyond

**Figure 5:**
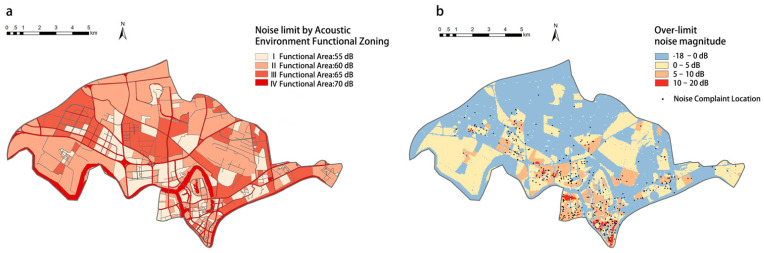
Comparison of monitored and complaint noise against the *National Standard* directions. (**a**) The acoustic environment functional zoning map based on the *National Standard*; (**b**) overlay of noise complaints with the difference between monitored noise magnitude and maximum noise limits as directed by the *National Standard*.

Practically, this study would help us to accurately infer the noise events that lead to complaints, i.e., people’s expressed dissatisfaction, thus contributing to more targeted and precise control of urban noise and improvement of the quality of the urban acoustic environment in general. Therefore, this study also has a direct policy implication. Comparing the acoustic environment functional zoning map of the study area as directed by the *National Standard* (Figure 5a) with the monitored noise magnitudes (Figure 5b background), we yield the blue areas where the physical noise level meets the standard, and areas with the other three colors where the physical noise exceeds the noise limit in the standard. 

The darker the color, the greater the magnitude of noise exceeding the standard. It can be seen that only about half of the areas meet the standard, which are basically water bodies and large areas of farmland or industrial areas. Further, by overlaying this map with the noise complaint points (Figure 5b foreground), it can be found that although most of the locations with noise complaints are in the areas that exceed the standard, there are also complaints in the areas that partially meet the standard. Moreover, the areas where the noise complaints are dense are not necessarily the areas where the standard is exceeded the most: there are also many complaints in the areas where the standard is exceeded only by 0 to 5 dB. These patterns illustrate that, on the one hand, the unsatisfactory level of enforcement of the *National Standard*; and on the other hand, even if the *National Standard* is fully complied with, noise complaints will still not be avoided. This finding again confirms that physical noise magnitude and people’s subjective tolerance of noise are not the same thing. Therefore, the introduction of the Social Sensing perspective in possible future revisions of the *National Standard*, with people’s perception rather than objective physical measures as the policy objective, is expected to better respond to the actual needs of people, and thus help shape a more satisfying urban environment.

Beyond the *National Standard*, there could be other lessons taken from the study. Rather than serving as direct basis for the local government to impose penalties on noise-producing or complaint-eliciting entities, the *National Standard* in China’s centralist system plays more of a role for higher-level governments to evaluate the governance performance of lower-level ones. The latter would create specific locally imposed measurements to solve noise-related problems if they feel the pressure from above, and this is the time when specific knowledge on the spatiotemporal pattern and occurrence mechanism of noise (complaints) may weigh in. For example, the road density variable does not enter the noise perception model. Although not directly supported by empirical evidence, we speculate that this is because of the local regulation on traffic noise. Ningbo City had issued the *Circular of the Ningbo Municipal People’s Government on the Implementation of the New Four Traffic Prohibitions within the Central City Area*, in which the prohibition of horn sounding was stipulated as: all kinds of motorized vehicles equipped with electric or gas horns are prohibited from sounding their horns in the central city area 24 h a day. In addition, in order to strengthen regulation, Ningbo’s traffic control department activated the illegal horn sounding capture system on urban roads on 7 April 2018, which has played a suppressive role in traffic noise in the central city area. If this hypothesis can be testified in future research with more empirical data then the imposition of such regulations could prove to be effective measures for suppressing traffic-related noise, which could serve as a useful lesson for other cities.

### 4.4. Limitations and Future Work

The research still has certain limitations. First of all, due to data availability issues, we were not able to include fine-grained noise monitoring data with longer time periods, nor nighttime noise monitoring data in the physical noise model. Although for stable spatiotemporal pattern and mechanism analysis, our assumption that the long-time noise pattern should be stable in an established urban area appears a fairly reasonable one, direct empirical evidence is after all much preferable for stronger findings. Also, for similar data availability reasons, we were not able to include census tract-level demographic and socioeconomic statistics data as explanatory variables for both models, which appears to have at least affected the explanatory power of the physical noise model. Overall, the missing of potentially influential data might have negatively affected the generality of the research, and is one issue subject to enhancement in future research.

Future research may also benefit from the introduction of other Social Sensing data sources of noise perception. For example, other research has utilized social media data on environmental noise to produce the “chatty map” [17] for urban areas. Such alternative Social Sensing data sources may prove good complement to our research, and they together may portray a more comprehensive perceptual acoustic landscape. Furthermore, one can also consider including physiological indicators that reflect noise’ health impacts, such as those recommended by the WHO [57], such that the physical-psychological-physiological effects of the noise environment can be investigated within one unified analytical framework to yield a more comprehensive understanding of the subject.

## 5. Conclusions

In this paper, we borrowed from environmental perception theories, and argued that the physical magnitude of urban noise and the perceived level of noise by urban residents are equally important in noise governance, and consequently established an analytical framework that integrates the two perspectives. Empirically, aside from new noise monitoring data that fills the spatiotemporal granularity gaps of official noise monitoring, we introduced the “12345” urban complaint hotline records as a proxy for the residents’ perceived noise levels, and constructed mechanistic models for physical magnitude and perceived seriousness of urban noise, respectively by taking the Jiangbei District of Ningbo City, China as an example. We found that the semantics of urban places, temporal rhythms of life, and population demographics significantly influenced people’s tolerance of noise, making the *perceived* noise problem map presenting a vastly different picture from the *physical* noise map as sensed by infrastructural sensors. We concluded that the existence of perceptual bias and behavioral preference effects in the noise perception process explained the difference, and that the existing National Acoustic Environmental Quality Standard does not adequately meet the needs of urban noise governance, and should be updated to reflect the perceptual aspect of the urban noise problem.

## Figures and Tables

**Table 1:** Variables in the physical noise magnitudes model.

Variable Category	Description	Max	Min	Average	Var
Population density	Population as recorded in the mobile phone signaling records/grid area	0.3118	0.0022	0.0359	0.0013
Road density	Road land area/grid area	0.40186	0	0.11873	0.00637
Urban functional mixture level	POI-based urban functional mixture index	1.19600	0	0.16200	0.05090
POIs	Food and beverage services POI count/grid area	0.21954	0	0.01369	0.00103
	Shopping service POI count/grid area	0.30000	0	0.02816	0.00271
	Office building POI count/grid area	0.02800	0	0.00088	0.00001
	Leisure and entertainment services POI count/grid area	0.20538	0	0.01014	0.00050
	Government and administration agencies POI count/Grid area	0.10800	0	0.00392	0.00015
	Financial services POI count/grid area	0.04400	0	0.00302	0.00004
	Tourist attractions POI count/grid area	0.05732	0	0.00116	0.00002
	Automobile services POI count/grid area	0.0675	0	0.0078	0.0001
	Street vending POI count/grid area	0.31179	0.00218	0.03588	0.00132

**Table 2:** Variables in the Perceived Noise Levels Model.

Variable Type	Variable	Code	Min	Max	Average	Var
Physical noise magnitude	Equivalent sound level	$L_{aeq}$	53.12067	72.76729	60.93731	5.60515
Place semantics	Percentage of residential land	res101	0.00000	0.96923	0.29357	0.09088
	Percentage of office building land	offices201	0.00000	0.29002	0.01030	0.00148
	Percentage of markets and shopping centers land	ma202	0.00000	0.60756	0.02508	0.00795
	Percentage of industrial land	indus301	0.00000	0.99219	0.21198	0.09322
	Percentage of bus station land	station402	0.00000	0.15977	0.00101	0.00013
	Percentage of government and administrative land	gov501	0.00000	0.29840	0.01330	0.00220
	Percentage of education and research land	edu502	0.00000	0.94566	0.05536	0.02634
	Percentage of medical land	hospital503	0.00000	0.29332	0.00393	0.00063
	Percentage of cultural and sports land	spo_cul504	0.00000	0.36356	0.01671	0.00353
	Percentage open space land	openspace505	0.00000	0.68544	0.05103	0.01493
	Percentage of agricultural land	farm601	0.00000	0.99009	0.13975	0.08121
	Percentage of water bodies	water602	0.00000	0.73339	0.05832	0.01888
	Functional mixture index- land use	S_landuse	0.01984	0.79269	0.38445	0.02942
Demographics—residents	Percentage under 18 years old	jz18	0.00676	0.28462	0.03651	0.00077
	Percentage 19–34 years old	jz19_34	0.20982	0.84677	0.41267	0.00982
	Percentage 35–49 years old	jz35_49	0.06048	0.44774	0.31169	0.00304
	Percentage 50–64 years old	jz50_64	0.04805	0.35027	0.18848	0.00255
	Percentage 65 years old and above	jz65	0.00314	0.12500	0.05065	0.00065
	Residential population mixture index	jz_S	0.39064	0.72707	0.64646	0.00335
Demographics—working population	Percentage under 18 years old	gz18	0.00000	0.21769	0.02571	0.00052
	Percentage 19–34 years old	gz19_34	0.15584	0.60222	0.41026	0.00741
	Percentage 35–49 years old	gz35_49	0.21088	0.51485	0.34679	0.00208
	Percentage 50–64 years old	gz50_65	0.07477	0.35714	0.18677	0.00288
	Percentage 65 years old and above	gz65	0.00000	0.09434	0.03047	0.00041
	Working population mixture index	gz_S	0.42823	0.59975	0.52681	0.00119
Demographics—visiting population	Percentage under 18 years old	df18	0.01170	0.05822	0.02345	0.00005
	Percentage 19–34 years old	df19_34	0.31643	0.55981	0.42250	0.00162
	Percentage 35–49 years old	df35_49	0.27330	0.44082	0.35304	0.00045
	Percentage 50–64 years old	df50_65	0.08791	0.24413	0.17039	0.00063
	Percentage 65 years old and above	df65	0.00935	0.06377	0.03062	0.00012
	Visiting population mixture index	df_S	0.48250	0.58399	0.53014	0.00036

**Table 3:** Summary of Physical Noise Model Results.

Variable	Coef.	St. Err.	*t*-Value	*p*-Value	(95% Conf	Interval)
Population Density	2.717	0.451	6.02	0.000 ***	1.827	3.606
Functional Mixture Index-POI	1.825	1.596	1.14	0.254	−1.322	4.971
Shopping Services	−0.077	0.022	−3.51	0.001 ***	−0.120	−0.034
Office Buildings	0.484	0.215	2.25	0.025 **	0.06	0.907
Leisure and Entertainment Services	0.097	0.048	2	0.046 **	0.002	0.193
Government and Administration Agencies	0.13	0.047	2.73	0.007 ***	0.036	0.223
Financial Services	−0.256	0.139	−1.85	0.067 *	−0.530	0.018
Tourist Attractions	0.268	0.147	1.83	0.069 *	−0.021	0.557
Food and Beverage Services	0.037	0.025	1.49	0.139	−0.012	0.087
Automobile Services	−0.014	0.026	−0.53	0.599	−0.065	0.038
Street Vending	0.002	0.01	0.22	0.823	−0.017	0.022
Road Density	−2.104	1.804	−1.17	0.245	−5.661	1.453
Constant	59.972	0.243	247.19	0	59.494	60.45
Mean dependent var	60.937		SD dependent var		2.373	
R-squared	0.443		Number of obs		210	
F-test	13.051		Prob > F		0	
Akaike crit. (AIC)	861.082		Bayesian crit. (BIC)		904.595	

Dependent Variable: $L_{aeq}$; *** *p* < 0.01, ** *p* < 0.05, * *p* < 0.1.

**Table 4:** Summary of the results of the noise complaint model (6:00–19:00) for the everyday-life-related type of noise.

Variable	Coef.	St. Err.	*t*-Value	*p*-Value	(95% Conf	Interval)
$L_{aeq}$ ***	0.016	0.006	2.81	0.005 ***	0.005	0.027
Res ***	0.345	0.051	6.75	0.000 ***	0.245	0.446
Slu **	0.194	0.089	2.19	0.030 **	0.019	0.369
JZa(19_34) **	−0.936	0.455	−2.06	0.041 **	−1.833	−0.038
JZb(35_49) **	−0.965	0.472	−2.05	0.042 **	−1.895	−0.035
JZc (50_64) **	−1.494	0.669	−2.23	0.027 **	−2.814	−0.174
GZb(35_49) **	0.845	0.330	2.56	0.011 **	0.193	1.496
Constant	−0.530	0.543	−0.98	0.330	−1.600	0.540
/Sigma	0.150	0.013			0.124	0.175
Mean dependent var	0.046		SD dependent var		0.091	
Pseudo R-squared	0.615		Number of obs		210.000	
Chi-square	65.478		Prob > chi2		0.000	
Akaike crit. (AIC)	58.974		Bayesian crit. (BIC)		89.097	

Dependent Variable: Noise Complaint Volume; *** *p* < 0.01, ** *p* < 0.05.

**Table 5:** Partial Marginal Effects of the Variables.

Variable	y*	*y|y > *0	*y*
$L_{aeq}$	0.016	0.005	0.006
Res	0.345	0.107	0.128
Slu	0.194	0.060	0.072
JZa (19_34)	−0.936	−0.290	−0.347
JZb (35_49)	−0.965	−0.299	−0.358
JZc (50_64)	−1.494	−0.463	−0.555
GZb(35_49)	0.845	0.262	0.314

## Data Availability

Restrictions apply to the availability of the complaint data. The data was obtained from Ningbo Bureau of Urban Management and are available from the authors with the permission of Ningbo Bureau of Urban Management. All other data presented in this study are openly available, please refer to the “2.1 Study Area and Data” section for detailed repositories or references of the data.

## Appendix A. The Acoustic Environment Functional Area Regulations in the National Acoustic Environmental Quality Standard (GB 3096-2008) and Rough Comparison with WHO Recommendations

**Table A1:** Environmental noise standards and ISO recommendations (Unit: Decibels). Source: National Acoustic Environmental Quality Standard (GB 3096-2008) [7]; New WHO Guidelines for Community Noise [57].

Acoustic Environment Functional Area	Area Description	$\mathrm{Daytime}/\mathrm{dB}\;L_{aeq}$	$\mathrm{Nighttime}/\mathrm{dB}\;L_{aeq}$	$\mathrm{Comparison}\;\mathrm{with}\;\mathrm{WHO}\;\mathrm{Recommendations}/\mathrm{dB}\;L_{aeq}$
Class 0	Rehabilitation area	50	40	30 for hospitial, ward rooms, treatment rooms
Category 1	Residential, medical and health care, culture and education, scientific research and design, administrative office areas	55	45	35–45 for indoor residential and education environments; 50–55 for outdoor living areas
Category 2	Commercial, financial and market trade-oriented areas	60	50	70–85 for commercial or public environments
Category 3	Industrial production, storage and logistics areas	65	55	70 for industrial environments
Category 4	The area within a certain distance on both sides of traffic arteries	70	55–60	85 for public addresses; 100 for public events

## Appendix B. Example of the “12345” Complaint Records

**Table A2:** Example of the “12345” Complaint Records. Source: Ningbo City Government.

ID	Date	Time	Complaint Content	Category	Long	Lat
201704369	29 April 2017	20:46	Near Wanda Plaza’s Gate on Yunfei Road, someone has built a stage where performance sound is too loud.	Everyday life	121.5266218	29.9184564
201707161	1 July 2017	20:57	Barbecue store near the Hongtang Baixin kindergarten recently set up night stalls that open all night and after five o’clock in the morning. very noisy.	Everyday life	121.5030490	29.938677
201804157	8 April 2018	10:00	The construction of the west side of New Seaview Garden disturbs the public and affects children’s rest during the college entrance examination	Construction	121.5390441	29.89676918
201906192	8 June 2019	23:48	Construction of Metro Line 4 in Double East Place of Cypress Garden disturbs the public	Construction	121.5288853	29.89494373

## Appendix C. Mobile Phone Signaling Data Classification Standards

**Table A3:** Classification Standards of the Mobile Phone Signaling Data. Source: China Unicom.

Class	Under 18 Years Old	19–34 Years Old	35–49 Years Old	50–64 Years Old	Over 65 Years Old
Resident population	Observation period: 21:00–8:00 of the next day; the number of seconds observed by the user daily during the observation period is accumulated monthly and ranked, and the highest ranked one is identified as the user’s place of residence.				
Working population	Observation time period: 9:00 to 17:00; the number of seconds observed during the observation time period on the user’s working day, accumulated monthly and ranked; the highest ranking is identified as the user’s working place.				
Visiting population	Short stay (30 min or more) at a place of residence or work nature where a person does not live or work is identified as a visiting record of the user.				

## Appendix D. Land Use Classification Table

**Table A4:** Land Use Classification Table. Source: [39].

	Primary Classification		Secondary Classification	Description
1	Residential land	101	Residential land	Residential areas
2	Commercial Land	201	Office Building	Office buildings, commercial buildings, etc.
	Commercial Land	202	Markets and shopping centers	Shopping centers, malls, commercial plazas, markets, etc.
3	Industrial Land	301	Industrial Land	Factories, industrial parks, etc.
4	Transportation Land	401	Road land	Roads at all levels
	Transportation Land	402	Bus Station	Bus station and outer square
5	Public Facility Land	501	Government and administrative land	Government land, administrative unit land
	Public Facility Land	502	Educational and Research Land	Land for schools and research institutions
	Public Facility Land	503	Medical Land	Hospital and nursing home land
	Public Facility Land	504	Cultural and Sports Land	Museums, large theaters, gymnasiums, cultural centers, etc.
	Public Facility Land	505	Open Space	Parks, squares, etc. at all levels
6	Production land	601	Farmland	Farmland
	Production land	602	Water bodies	Water bodies
